# Circulating metabolites as potential biomarkers for the early detection and prognosis surveillance of gastrointestinal cancers

**DOI:** 10.1007/s11306-023-02002-0

**Published:** 2023-04-04

**Authors:** Guodong Song, Li Wang, Junlong Tang, Haohui Li, Shuyu Pang, Yan Li, Li Liu, Junyuan Hu

**Affiliations:** 1 The Second Hospital of Tianjin Medical University, No 23. Pingjiang Road, Hexi District, 300211 Tianjin China; 2 Metanotitia Inc, No 59. Gaoxin South 9Th Road, Yuehai Street, Nanshan District, Shenzhen, 518056 Guangdong China

**Keywords:** Metabolomics, Circulating metabolites, Gastric cancer, Colon cancer, Early detection, Biomarker, Surveillance

## Abstract

**Background and aims:**

Two of the most lethal gastrointestinal (GI) cancers, gastric cancer (GC) and colon cancer (CC), are ranked in the top five cancers that cause deaths worldwide. Most GI cancer deaths can be reduced by earlier detection and more appropriate medical treatment. Unlike the current “gold standard” techniques, non-invasive and highly sensitive screening tests are required for GI cancer diagnosis. Here, we explored the potential of metabolomics for GI cancer detection and the classification of tissue-of-origin, and even the prognosis management.

**Methods:**

Plasma samples from 37 gastric cancer (GC), 17 colon cancer (CC), and 27 non-cancer (NC) patients were prepared for metabolomics and lipidomics analysis by three MS-based platforms. Univariate, multivariate, and clustering analyses were used for selecting significant metabolic features. ROC curve analysis was based on a series of different binary classifications as well as the true-positive rate (sensitivity) and the false-positive rate (1-specificity).

**Results:**

GI cancers exhibited obvious metabolic perturbation compared with benign diseases. The differentiated metabolites of gastric cancer (GC) and colon cancer (CC) were targeted to same pathways but with different degrees of cellular metabolism reprogramming. The cancer-specific metabolites distinguished the malignant and benign, and classified the cancer types. We also applied this test to before- and after-surgery samples, wherein surgical resection significantly altered the blood-metabolic patterns. There were 15 metabolites significantly altered in GC and CC patients who underwent surgical treatment, and partly returned to normal conditions.

**Conclusion:**

Blood-based metabolomics analysis is an efficient strategy for GI cancer screening, especially for malignant and benign diagnoses. The cancer-specific metabolic patterns process the potential for classifying tissue-of-origin in multi-cancer screening. Besides, the circulating metabolites for prognosis management of GI cancer is a promising area of research.

**Supplementary Information:**

The online version contains supplementary material available at 10.1007/s11306-023-02002-0.

## Introduction

Cancer of the gastrointestinal (GI) tract, including the esophagus, stomach, gallbladder, small intestine, colon, and rectum, is very common. Therein, colorectum cancer and gastric cancers are the two most aggressive types of GI cancer contributing to cancer cases and deaths across the world (Arnold et al., 2020). According to global cancer statistics in 2020, there were an estimated 1.9 million and 1.1 million new cases and 0.9 million and 0.8 million new deaths of colorectum and gastric cancer, respectively (Sung et al., 2021). These two cancers are among the top five worldwide in terms of morbidity and mortality in both males and females. Colorectal cancer occurs when malignant cells form in colon and rectum tissues. Hence, colorectal cancer can be further classified into colon cancer (CC) and rectal cancer (RC). CC is a more malignant and leading cause of cancer-related death than RC (Sung et al., 2021). Gastric cancer (GC) forms in the tissues that line the stomach. Because the digestive tract is a continuous system, many GI cancers cause the same clinical symptoms, such as blood in the stool, vomiting, and unexplained weight loss (https://hillman.upmc.com/cancer-care/colorectal-gi/symptoms-diagnosis). As these clinical symptoms often do not appear until the advanced stage of GI cancer, some specific tests are required to facilitate diagnosis. Commonly, endoscopy is used to determine tumor locations, and biopsy is used to clinically confirm the cancer types. However, these “gold standard” approaches are invasive, have low compliance, and are usually appropriate for advanced stages, which normally means chemotherapy and/or surgery are needed. The occurrence of cancer can extend over a long period comprising numerous stages (Bert et al., 2013). To date, most cancers can be cured by surgery if performed at the early stage before tumor cells have metastasized to distant sites. Once the tumor has developed and is considered late stage, treatment is quite challenging, and prognosis is poor. Thus, earlier screening and diagnosis are vital for reducing tumor-related deaths. As reported by Miller et al., more than half of cancer patients survived well in the past ten years because of advances in early detection and treatment (Miller et al., 2019a). Equivalently, cancer prognosis is important for patient survival in estimating cancer development and improving clinical management. There are precise methods for monitoring cancer recurrence, spread, and stages after surgery, e.g., computerized tomography(CT) and magnetic resonance imaging (MRI), but these have several limitations, including sensitivity, availability, cost, and radiation risk (Martins et al., 2021; Saluja et al., 2018; Yip et al., 2015).

The development of effective techniques for cancer screening and surveillance is one of the foremost challenges encountered in modern cancer research. In recent years, methods for detecting cancer in a slightly- or non-invasive way have been developed to improve screening and exams. For example, the blood tumor markers (e.g., carcinoembryonic antigen, carbohydrate antigen 19-9 and cancer-related antigen 72-4) have been widely used in clinical diagnosis. However, they generally have low sensitivity and specificity (Jung et al., 2014). Another revolutionary approach, namely, “liquid biopsy”, exhibits significant potential for monitoring cancer evolution and treatment response in real time (Martins et al., 2021). The detection of circulating transcripts and tumor cells in blood could provide accurate diagnostic results; however, sensitivity is an issue because these markers are found in only small amounts at the early stage of cancers, often below the detection limit or making it difficult to measure any alterations (Adashek et al., 2021; Chetan et al., 2014; Cohen et al., 2017). Alternatively, “omics” technologies enable us to study the cancer-related alterations at not only a genetic level but also at proteomic and metabolomic levels with high sensitivity. Among these techniques, metabolomics is a promising approach for cancer biomarker discovery, as the reprogramming of cellular metabolism is one of the hallmarks of cancer, and metabolomics as the endpoint of “omics” cascades could reflect perturbations in all biological activities with an amplified way (Hanahan, 2022; Hasin et al., 2017; Ni et al., 2014). Numerous metabolomics studies have been conducted to screen the biomarkers of cancers, whereas there are common metabolic features among different cancer types, such as the Warburg effect and the reprogramed citrate (TCA) cycle, which result in challenges regarding differential diagnosis. For example, GC and CC, both being digestive tract cancers, share certain characteristics, and thus inevitably exhibit some indistinguishable metabolic features, resulting in difficulties for differential diagnosis. For instance, it has been found that GC and CC share a common pattern in that they differ in cysteine and methionine metabolism, arginine and proline metabolism, the citrate cycle (TCA cycle), and glycolysis or gluconeogenesis (Ren et al., 2021; Yu et al., 2020). In addition, relevant research has demonstrated that tumor resection surgery events can distinctly change the metabolic characteristics in multiple cancer types. For example, curative surgery significantly changes the level of γ -linolenic acid in CRC and alanine, arginine, and hypoxanthine in GC (Jung et al., 2014; Zhuang et al., 2022).

To explore the potential role of circulating metabolites in the diagnosis and surveillance of GI cancers, we investigated the plasma metabolomics of GC, CC, and non-cancer patients enrolled in the Second Hospital of Tianjin Medical University (Tianjin, China). GC and CC samples can be adequately distinguished from non-cancer samples on a global metabolomic level. Many metabolites differed between GC and CC samples, and this can be used to diagnose and distinguish cancerous and non-cancer individuals. In addition, metabolites that are distinctly different between GC and CC samples can be applied in cancer-type identification, e.g., PE 40:8e, PE 40:3, PE 36:1p, PC 42:1, and (2α, 3α, 5ξ, 9ξ)-2,3-dihydroxyurs-12-en-28-oic acid. We further investigated the metabolic differentiations between before and after surgery. The metabolites significantly altered by surgery can be used as surveillance biomarkers.

## Materials and methods

### Participants

The cohort is a subset population from our previous study. Participants were screened according to the inclusion and exclusion criteria as previously described (Shi et al., 2022). Subjects were excluded if one of the exclusion criteria was met or if they failed in the sample quality check. Informed consents were obtained from all patients, and ethical approval was received from the Ethics Committee of The Second Hospital of Tianjin Medical University (China). Participating patients had a diagnosis of gastric or colon cancer, and patients without cancer or with inguinal hernia served as controls. Demographic information was collected, such as age, chronic disease, and smoking history. Five and eight patients with gastric and colon cancer, respectively, underwent surgical resection during this study.

### Sample collection and preparation

Blood samples were collected from patients in a fasted state and processed as previously described (Shi et al., 2022). Briefly, 5 mL of peripheral blood was collected and centrifuged at 4 °C (3800 rpm, 10 min), and the supernatant was harvested for metabolite extraction. Samples (50 uL) were mixed with 700 uL MTBE buffer containing 0.45 μg/mL of gibberellic acid A3, 1 μg/mL of ^13^C sorbitol, and 0.45 μg/mL of PC (17:0/14:1) as internal standards (Salem et al., 2016). The addition of 350 uL methanol/water (v/v, 1:3) was used for phase separation. The lipophilic and hydrophilic phases were separately collected for lipidomics and metabolomics analysis, respectively. Three types of quality control (QC) samples were prepared following the same procedure as above described, including a pooled 50% of randomly selected plasma samples (QC_bio_), a mixture of chemical standards (QC_mix_) (Shi et al., 2022), and only solvents (QC_blank_).

### Analytical platforms and sample detection

Lipids were analyzed by LC–MS, and polar metabolites were detected using both GC–MS and LC–MS. Liquid chromatographic separation was carried out on a Waters ACQUITY (Milford, MA) ultra-performance liquid chromatography (UPLC) system interfaced with a Thermo Fisher Q-Exactive (Bremen, Germany) mass spectrometer with an electrospray ionization (ESI) source. Polar phase was resolved in 200 μL of water and lipophilic fraction was resuspended in 200 μL of acetonitrile/isopropanol (v/v, 7:3). 3 μL of supernatant was transferred to a Waters ACQUITY FTN autosampler with the temperature set to 10 °C. Data were acquired both in positive and negative modes. An Agilent 7890B gas chromatograph with an Rxi^®^-5SilMS GC column (30 m, 0.25 × 30 mm, 0.25 μm) coupled to a Pegasus BT time-of-flight (TOF) mass spectrometer (Leco Corp., St. Joseph, MI, USA) with an electron ionization (EI) source was used for derivatized metabolite detection. The carrier gas was high-purity helium at a flow rate of 1 mL/min. The initial temperature was set at 50 °C for 2 min and increased by 1 °C/ min until up to 330 °C. The interface and ion source temperatures were adjusted to 280 and 250 °C, respectively. The detector voltage was maintained at 1.2 kV with standard 70 eV EI parameters. The analytical conditions and parameters for data acquisition were detailed in our previous methodology study (Shi et al., 2022).

### Data extraction and compound identification

LC–MS chromatograms were processed using Metanotitia Inc. in-house developed software *PAppLine*™ and refined as described by Giavalisco et al. (Giavalisco et al., 2009, 2011). The peaks were extracted from each chromatogram with a baseline correction to remove the noise, and then matched across samples and aligned the retention time (RT). The identified features were annotated using the Metanotitia Inc. library, Ulib^MS^. Polar metabolites were identified by a six-thousand sub-library. The lipids were annotated by a 1700 lipophilic library. Chromatograms obtained from GC–MS analysis were submitted to ChromaTOF (version 5.40) and refined and annotated using TargetSearch (R version 4.0.3) based on the Fiehn reference libraries (Cuadros-Inostroza et al., 2009; Kind et al., 2009). Peak detection, retention time alignment and calculated as the RT shift throughout all batches for each FAME internal standard, and mass spectral comparison with FAME standards, and finally quantified by the peak intensity. More details are described in our previous methodological paper (Shi et al., 2022).

### Data processing and statistical analysis

All metabolic features from the GC–MS and LC–MS platforms with an overall coverage below 50% were filtered and the rest further normalization using Python 3.7.6 Anaconda Edition (Anaconda Software Distribution, 2016). The missing values were divided into three groups, such as abundance > 50%, abundance < 50% and a mean intensity > 1E5, and abundance < 50% and a mean intensity < 1E5. Each situation was imputed with median ± 5% random noise, limit of detection (LOD) ± 5% random noise, and 0, respectively. RobustScaler from Scikit-learn package in Python was used to scale the data while reducing the impact of outliers comparing to other methods. The batch effect was corrected by a non-linear autoencoder implemented in Normalization Autoencoder (NormAE) package (Rong et al., 2020). Briefly, this deep learning method embedded original high-dimensional data into low-dimensional space. Data reconstruction was performed subsequently to transform those representations in low-dimensional space back into high-dimensional data to remove the inter-batch effect. Furthermore, the intra-batch effect was identified and removed by the autoencoder which was optimized by adversarial regularization and discriminators. The annotated metabolites were analyzed using both univariate and multivariate approaches as well as machine learning. The heatmaps of differentiated the value of metabolites between groups by colors and shades, while the dendrogram with weighted linkage method to use for calculating clusters reveals the relatedness of or dissimilarities between groups. The significantly changed metabolites of GC or CC samples compared with NC were calculated by *t*-test with cutoff parameters of |Log2(foldchange)|> 0.25 and (BH-adjusted) *p* value < 0.05. Pathway enrichment analysis was based on MetaboAnalyst 5.0 (https://www.metaboanalyst.ca/). Differentiated metabolic were selected by a tree-based algorithm XGBoost with the help of the Python package scikit-learn (Stamate et al., 2019), and the significance was calculated by a *t*-test (VIP > 1, (BH-adjusted) *p* value < 0.05). Then, the network was visualized by VANTED (http://vanted.sourceforge.net/#ui-tabs-4). The ROC curves were based on a series of different binary classifications (demarcation value or determination threshold) as well as the true-positive rate (sensitivity) and the false-positive rate (1-specificity). The diagnostic performance was ranked, and the top five metabolites of each pair were displayed.

## Results

### Cohort characteristics

In this study, 81 participants were recruited, which included 37 gastric cancer (GC), 17 colon cancer (CC), and 27 non-cancer (NC) patients (Table 1). Of these, five and eight cancer patients (GC and CC) underwent surgical treatment. Therefore, a total of 94 plasma samples were collected, i.e., 45, 25, and 27 samples collected from the GC, CC and NC groups, respectively. In this case–control study, there were significant differences in female percentage and smoking between the cancer and non-cancer groups, which allowed us to determine the source of potential bias in metabolomics outcomes. However, there were no obvious differences between the GC and CC groups (Table 1), which allowed us to conduct a comparative analysis of the GC and CC groups.

**Table 1:** Baseline characteristics of participants

		Non-cancer (NC)	Gastric cancer (GC)	Colon cancer (CC)	*p* value
n (after surgery sample available)		27	37 (5)	17 (8)	
Gender (%)	Female	3 (11.1)	14 (37.8)	8 (47.1)	0.02
	Male	24 (88.9)	23 (62.2)	9 (52.9)	
Mean age (SD)		62.2 (17.0)	67.2 (9.9)	67.8 (14.7)	0.27
Diabetes	Yes	1 (3.7%)	6 (16.2%)	2 (11.8%)	0.29
Hypertension	Yes	8 (29.6)	17 (37.8%)	10 (58.8%)	0.15
Current smoking	Yes	3 (11.1%)	16 (43.2%)	5 (29.4%)	0.02

Values are mean (SD) or %

Age of continuous variables were analyzed by one-way ANOVA, the Chi-square test was used for gender, history of diabetes mellitus, history of hypertension, and current smoker.

### MS-based metabolite identification

**Fig. 1:**
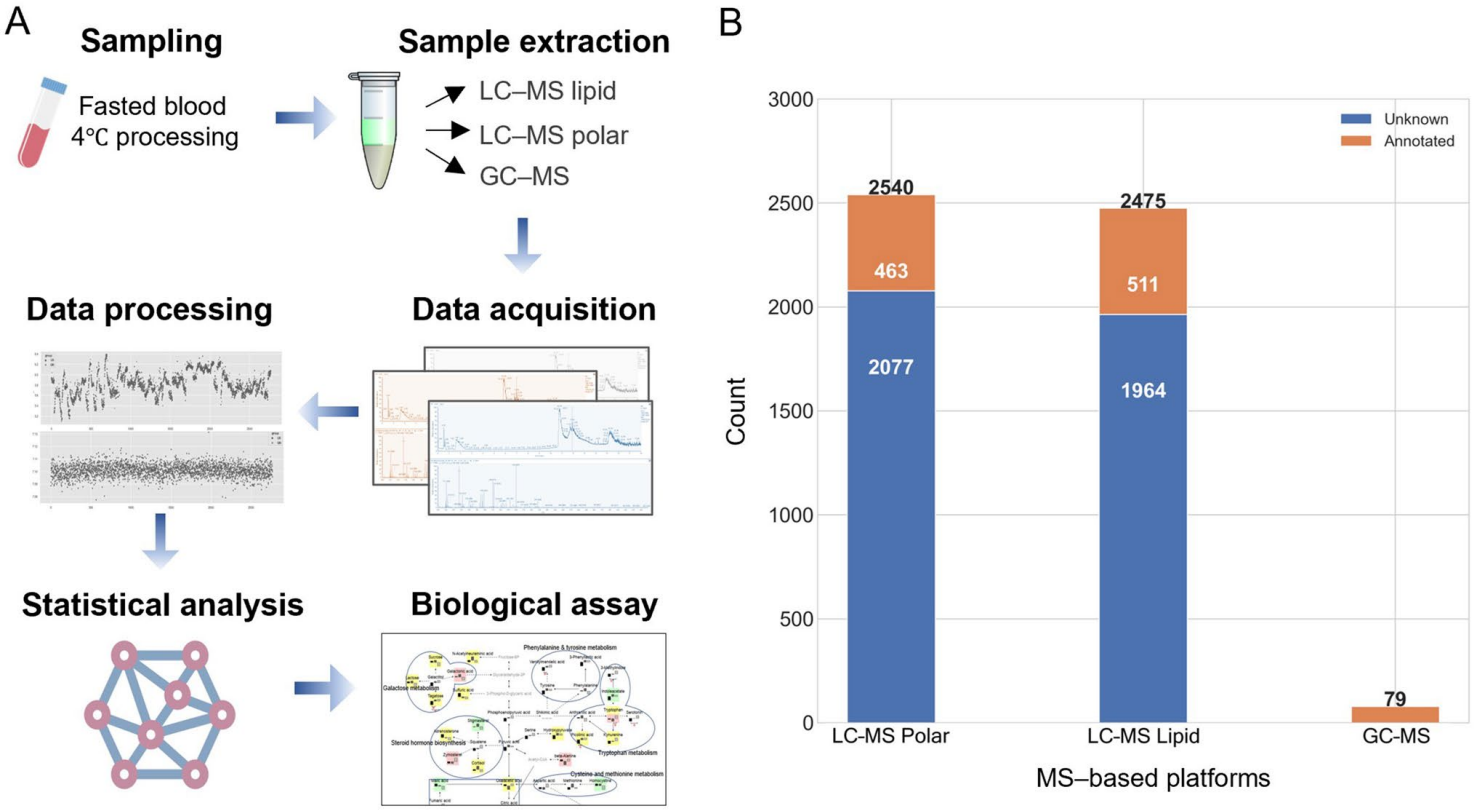
MS-based metabolomics and performance. **A** Workflow of the MS-based metabolomics. More details are described in a previous study (Shi et al., 2022). **B** Stacked chart of the metabolic features from the LC–MS polar, LC–MS lipid, and GC–MS platforms. The features of LC–MS platforms were obtained using both positive and negative ionization modes. Orange: annotated metabolites; Blue: unknown features

A total of 94 plasma samples were analyzed on three MS-based platforms according to the untargeted metabolomic method as previously described, and 2540, 2475 and 79 metabolic features were obtained from each (Fig. 1; Shi et al., 2022). Subsequently, 463, 511 and 79 features from the LC–MS polar, LC–MS lipid and GC–MS platforms, respectively, were annotated, and the rest remain unknown (Fig. 1B).

### Metabolomic differentiation of gastric cancer and colon cancer

**Fig. 2:**
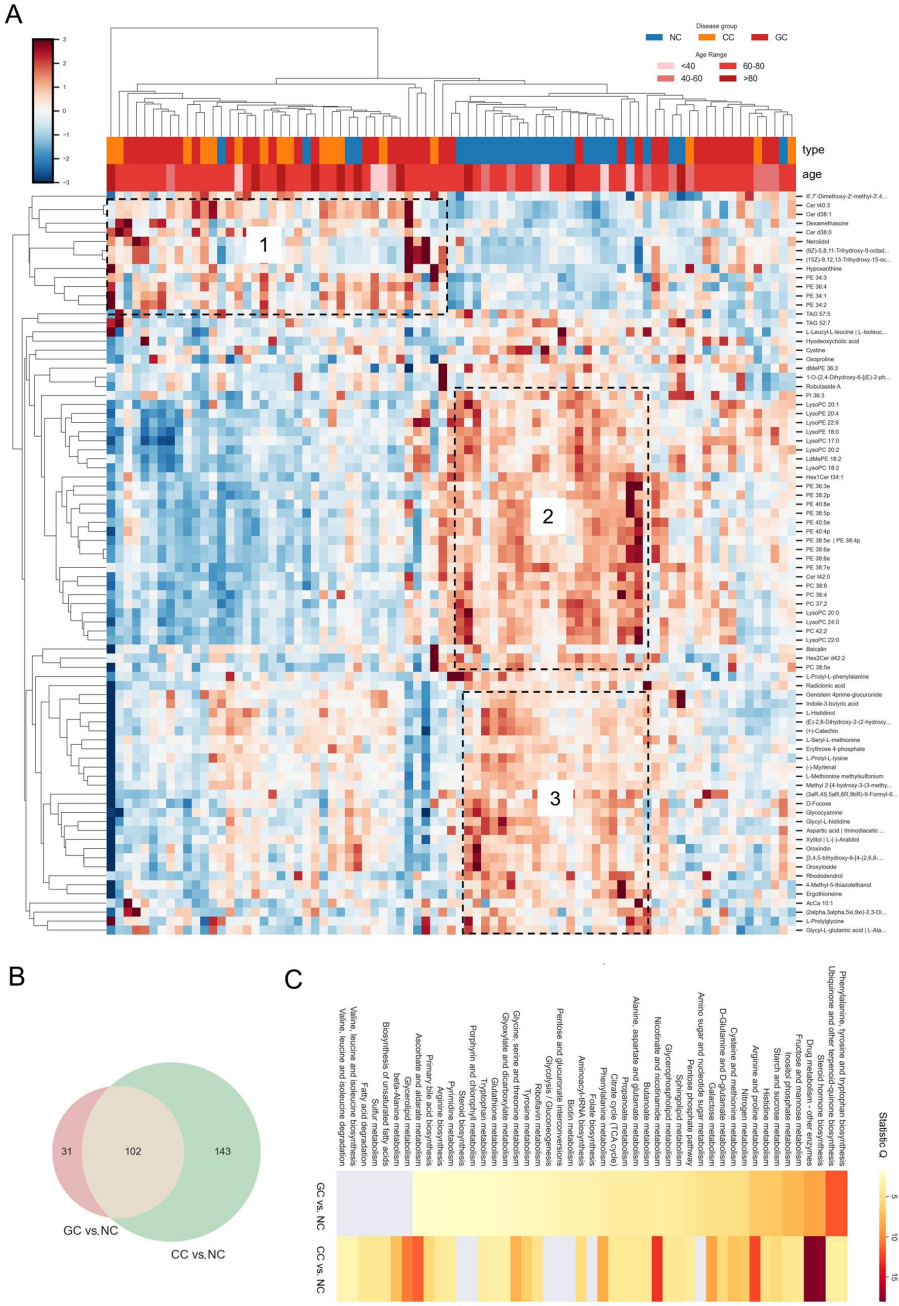
Metabolomic differentiation of gastric and colon cancer samples compared with non-cancer samples. **A** Heatmap of metabolites that differed between the cancer and non-cancer groups (Source data_1). Cluster method: weighted. The obviously changed metabolite clusters are highlighted in the three black dashed boxes. **B** Venn diagram of significantly changed metabolites of GC and CC samples compared with NC. |Log2(foldchange)|> 0.25, *p* value < 0.05 (Source data_2). **C** Pathway enrichment analysis of significantly changed metabolites in a comparison of the GC and CC groups with the NC group. Visualization was constructed using MetaboAnalyst 5.0 (https://www.metaboanalyst.ca/). GC: gastric cancer; CC: colon cancer; NC: non-cancer

Clinical blood tests for cancer must have high specificity to avoid false positive results which may lead to unnecessary follow-up procedures and anxiety. In this study, cancer and non-cancer samples exhibited satisfactory classification according to the metabolite clustering (Fig. 2A). Three cluster metabolites (in the black dashed boxes) obviously changed between the cancer and non-cancer groups. Cluster 1 mainly contained lipid ceramides (Cer) and PE, which were upregulated in the cancer samples. On the contrary, cluster 2 and 3 metabolites displayed relatively high levels in non-cancer samples, and consisted of lysolipids, PE and diverse polar metabolites (Fig. 2A). These results indicate that GC and CC can be sufficiently distinguished from NC at the metabolic level.

Although the GC and CC groups were not well distinguished by classification, they had obvious cancer-specific metabolic patterns when separately compared with the NC group (Fig. S1). CC and NC samples shown clearer classification in comparison with GC and NC. Lysolipids and PE mainly contributed to the classification. Moreover, a larger body of metabolites was significantly different in CC samples (Fig. 2B). Compared with NC samples, 245 and 133 metabolites were significantly altered in the CC and GC samples, respectively, wherein, 102 metabolites were commonly changed in both cancer groups (Fig. 2B). Pathway enrichment analysis of these common differentiated metabolites showed they mainly belong to steroid hormone biosynthesis, cysteine and methionine metabolism, arginine and proline metabolism, galactose metabolism, amino acids, and purine metabolism pathways (Fig. 2C; Fig. S2). Pathways that were found to be prevalent between the GC and CC groups with different levels of enrichment, included ubiquinone and other terpenoid-quinone biosynthesis, phenylalanine, tyrosine, and tryptophan biosynthesis, histidine metabolism, starch and sucrose metabolism, fructose and mannose metabolism, and inositol phosphate metabolism in GC samples, whereas in the CC samples, the prevalent pathways were nicotinate and nicotinamide metabolism, glycine, serine, and threonine metabolism, aminoacyl-tRNA biosynthesis, ascorbate and aldarate metabolism, glycerolipid metabolism, nitrogen metabolism, phenylalanine metabolism, tyrosine metabolism, and β-alanine metabolism. Metabolites that vary uniquely between GC and CC samples can be used for clinical purposes.

### Differential metabolites have a good diagnostic ability

**Fig. 3:**
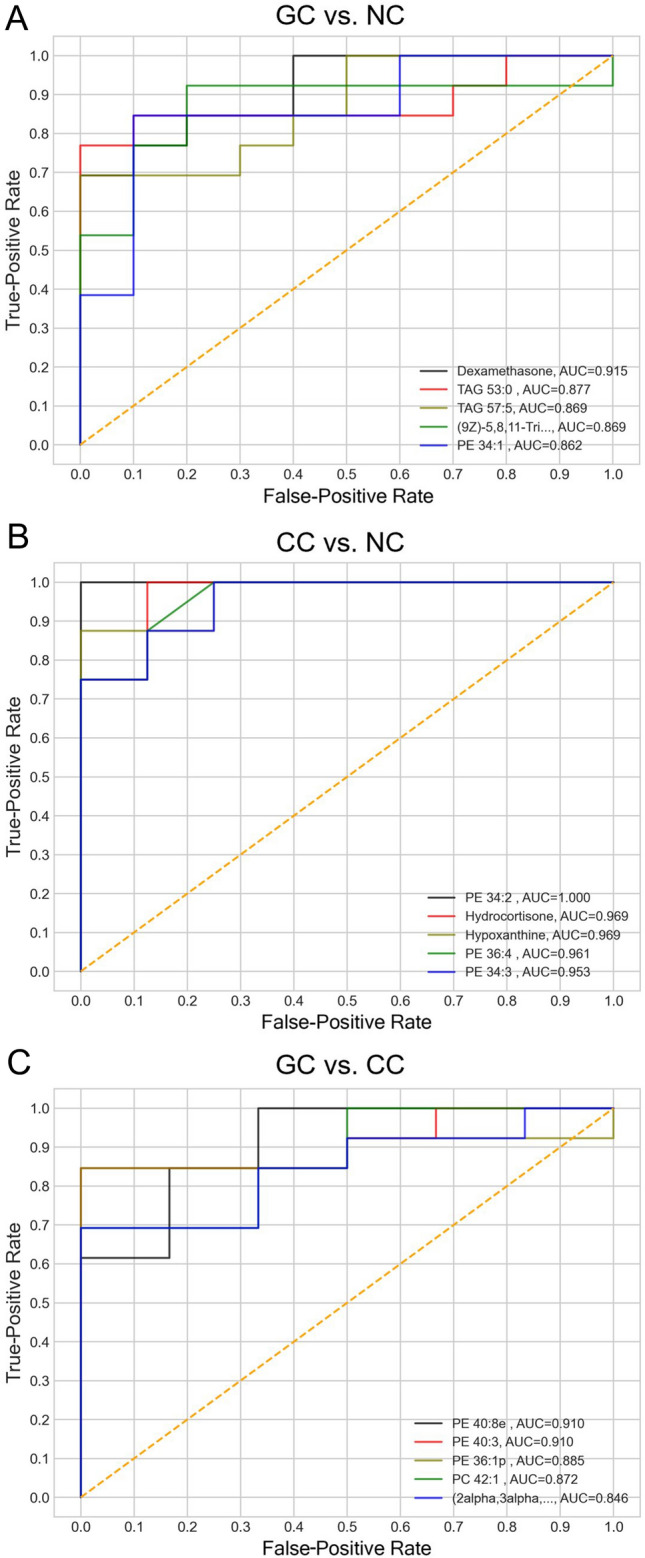
ROC curve analysis of metabolites in the different groups. ROC curve analysis of differential metabolites between (**A**) groups GC and NC, (**B**) groups CC and NC, and (**C**) groups GC and CC. The analyzed metabolites included dexamethasone, TAG 53:0, TAG 57:5, (9Z)-5,8,11-trihydroxy-9-octadecenoic acid, and PE 34:1; PE 34:2, hydrocortisone, hypoxanthine, PE 36:4, and PE 34:3; and PE 40:8e, PE 40:3, PE 36:1p, PC 42:1, and (2α, 3α, 5ξ, 9ξ)-2,3-dihydroxyurs-12-en-28-oic acid. GC: gastric cancer; CC: colon cancer; NC: non-cancer. The ROC curves were based on a series of different binary classifications (demarcation value or determination threshold) as well as the true-positive rate (sensitivity) and the false-positive rate (1-specificity)

To further screen the potential biomarkers for GI cancer diagnosis, we performed ROC analysis comparing the cancer and non-cancer groups. The top five diagnostic metabolites of each pair are displayed in Fig. 3. Dexamethasone, TAG 53:0, TAG 57:5, (9Z)-5,8,11-trihydroxy-9-octadecenoic acid, and PE 34:1 demonstrated good diagnostic ability in the groups of GC and NC (Fig. 3A). In the CC and NC groups, PE 34:2, hydrocortisone, hypoxanthine, PE 36:4 and PE 34:3 exhibited excellent diagnostic ability, with an AUC that exceeded 0.95 (Fig. 3B). Between the two cancer groups GC and CC, a group of phospholipids, including PE 40:8e, PE 40:3, PE 36:1p and PC 42:1, and (2α, 3α, 5ξ, 9ξ)-2,3-dihydroxyurs-12-en-28-oic acid had good diagnostic results with a mean AUC value that exceeded 0.88 (Fig. 3C). This suggests that the discriminated metabolites between the cancer and control groups have the potential to be developed as biomarkers for GI cancer detection and cancer-type identification.

### Metabolic differences before and after surgery

**Fig. 4:**
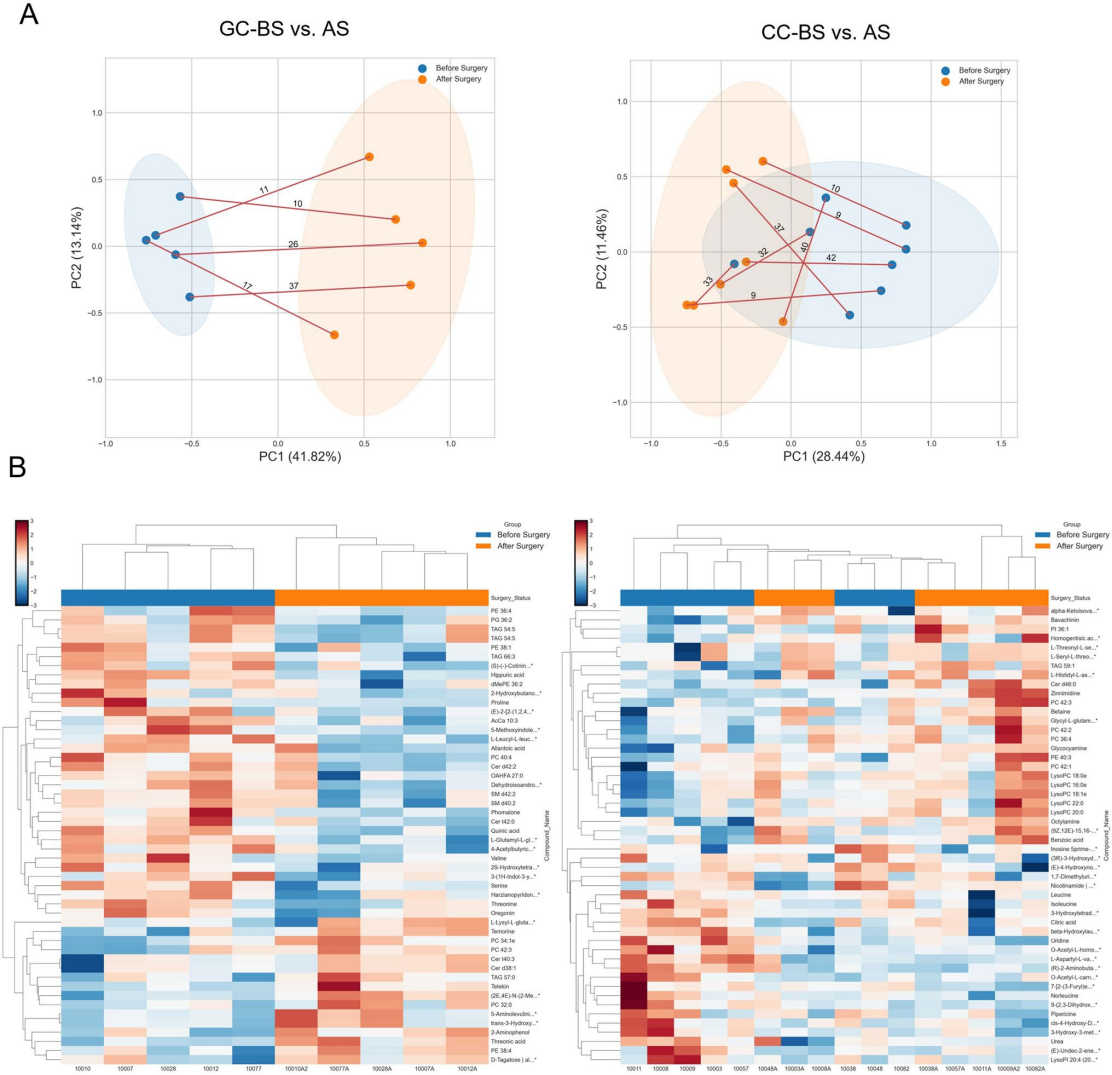
Metabolic differences before and after surgery in gastric and colon cancers from identical subjects. **A** PCA of metabolic profiles. Identical subjects are indicated using red connecting lines with the time interval (days). **B** Heatmap of the top 50 metabolites (Source data_3). Blue: before surgery; Orange: after surgery. GC: gastric cancer; CC: colon cancer; BS: before surgery; AS: after surgery

**Fig. 5:**
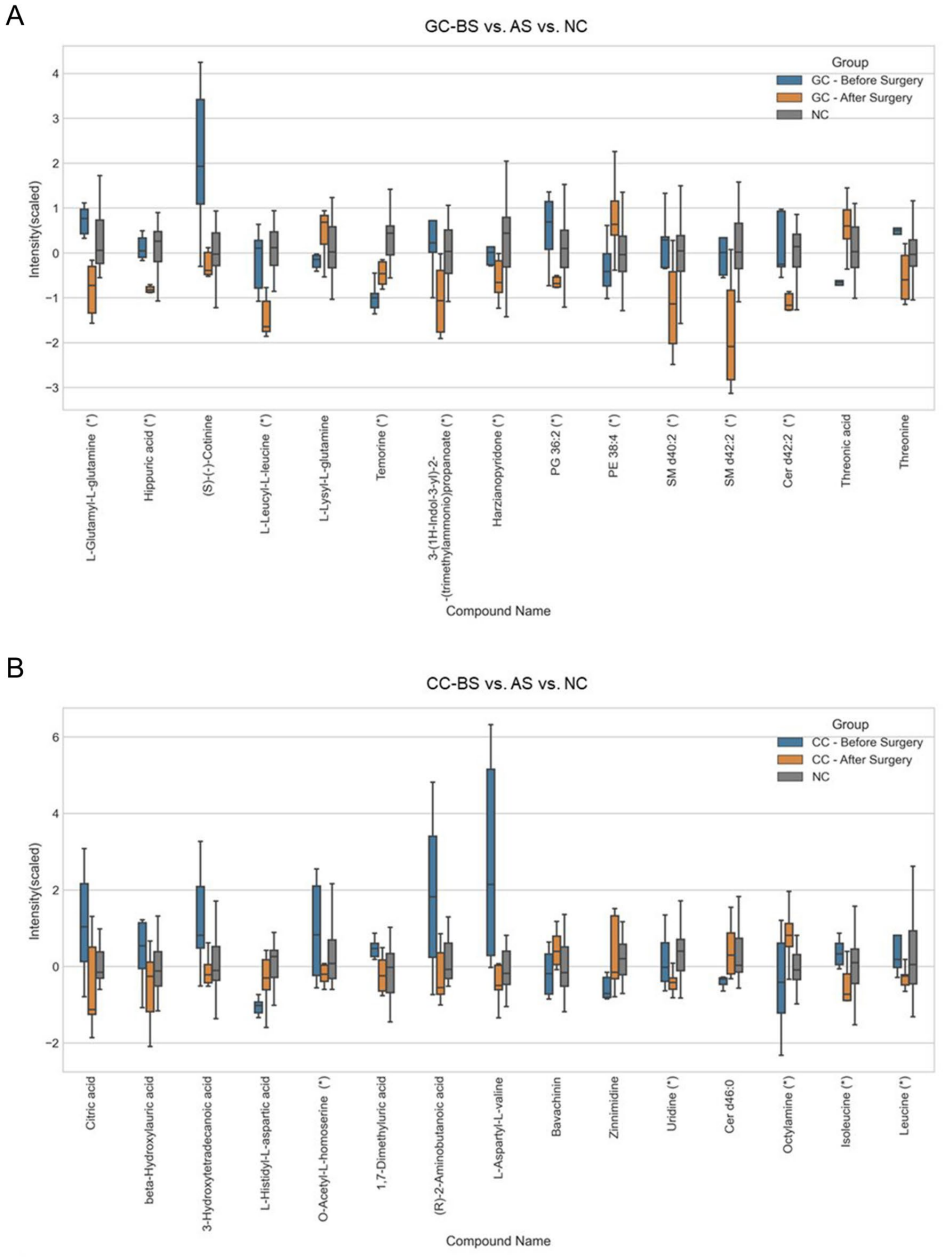
Differentiated metabolites before and after surgery. Box plots of significantly changed metabolites after the surgery of GC (**A**) and CC (**B**) patients and compared with NC control. |Log2(foldchange)|> 0.25, *p* value < 0.05. (*) indicates the significant difference between post-surgery and NC control, *p* value < 0.05. y axis: scaled intensity. Blue: before surgery (BS); Orange: after surgery (AS); GC: gastric cancer; CC: colon cancer; NC: non-cancer patient control. Source data are provided as Source data_4

The curative resection of gastrointestinal cancers remains a cornerstone in clinical management, but it is often challenging and has a poor prognosis. Moreover, current tools for assessing prognosis, determining treatment and surveillance, and for the early detection of recurrence lack sensitivity (Hsu et al., 2022; Saluja et al., 2018). To explore the potential of metabolic markers for post-surgical and personalized management, we characterized the plasma metabolomics of identical subjects before and after surgery. As estimated, the surgical resection of solid tumors dramatically affected the plasma metabolomic signatures of both GC and CC patients (Fig. 4). Metabolic patterns differed before- and after-surgery, particularly in GC subjects. The first principal component (PC1) separated the before- and after-surgery groups, explaining 41.82% and 28.44% of the variations in GC and CC cases, respectively. PC2 was mainly explained by the variability among the individuals (i.e., 13.14% of GC and 11.46% of CC) (Fig. 4A), wherein lipids largely contributed to the discrimination in the GC group, whereas lysolipids and polar metabolites contributed mostly to variations in the CC group (Fig. 4B). Furthermore, 15 metabolites were significantly altered in each case (Fig. 5), i.e., the levels of l-glutamyl-l-glutamine, hippuric acid, (S)-(–)-cotinine, l-leucyl-l-leucine, 3-(1*H*-indol-3-yl)-2-(trimethylammonio) propanoate, harzianopyridone, PG 36:2, SM d40:2, SM d42:2, Cer d42:2, and threonine were relatively high before GC surgery, and the levels of citric acid, beta-hydroxylauric acid, 3-hydroxytetradecanoic acid, O-acetyl-l-homoserine, 1,7-dimethyluric acid, (R)-2-aminobutanoic acid, l-aspartyl-l-valine, uridine, isoleucine, and leucine were relatively high before CC surgery; conversely, the levels of PE 38:4, threonic acid, l-lysyl-l-glutamine, and temorine were relatively high after GC surgery, and the levels of l-histidyl-l-aspartic acid, bavachinin, zinnimidine, Cer d46:0, and octylamine were relatively high in post-surgery CC. The significant metabolites after surgery, especially which returned to near normal levels (no significant difference compared with NC sample), can be developed as surveillance biomarkers, such as (*S*)-(–)-cotinine, l-lysyl-l-glutamine, threonic acid and threonine in GC samples, and 10 candidates in CC subjects (Fig. 5).

## Discussion

Digestive malignant neoplasms are the most common cause of cancer death worldwide. One reason why radically curing gastrointestinal cancer is very difficult is that the discovery often occurs when the cancer is at an advanced stage. Moreover, most of the clinical symptoms associated with GI cancers do not manifest cancer-type specificity until the late stages. Therefore, many researchers have attempted to find biomarkers for early and accurate detections of GI cancer by various approaches, such as transcriptomic, proteomics and metabolomics techniques. Here, we have attempted to identify metabolic features for GI cancer screening, tissue-of-origin identification, which is an inevitable issue in multi-cancer screening, and the potential for prognostic assessment. In this work, the case–control study is based on the plasma metabolomics of two GI cancers, gastric cancer (GC) and colon cancer (CC), as well as non-cancer control. When taking a global view, GC and CC are seen to have distinct metabolic patterns compared with non-cancer. This indicates that plasma metabolomics is a promising tool for the detection of GI cancers. Furthermore, the differential metabolic alterations of GC or CC can allow these cancer types to be distinguished. We also determined the plasma metabolomics before and after surgery. The significantly changed metabolites can serve as biomarkers for prognosis surveillance.

Considering the importance of changing metabolites in cancer progression, metabolomics is an emerging and promising technology for cancer research. The commonly utilized analytical techniques for GI cancer studies including GC–MS, LC–MS and especially NMR (Gao et al., 2023; Nannini et al., 2020; Yu et al., 2020). As summarized by Giulia Nannini et al., there are nine most significant metabolites identified from GI cancer bio-fluids via NMR. For instance, 3-hydroxybutyric acid and tyrosine were widely identified in blood, and acetate, butyrate and leucine were commonly detected in fecal water of CRC patients (Nannini et al., 2020). Another review article of GI cancer metabolomics was conducted by Zhenxing Ren et al. (Ren et al., 2021). The most significantly enriched pathways in GC samples are aminoacyl-tRNA biosynthesis, arginine biosynthesis, BCAA biosynthesis, tryptophan metabolism, and alanine, aspartate and glutamate metabolism (visualized by MetaboAnalyst 5.0 (https://www.metaboanalyst.ca/), −log(*p*) > 4.5). The most reported metabolites from CRC studies are glutamic acid, phenylalanine, alanine, lactic acid, cysteine, tyrosine, and tryptophan. They also compared the altered metabolic pathways for GI cancers. The shared pathways have different effects on different cancer types, for instance, phenylalanine, tyrosine, tryptophan biosynthesis ranks as GC < CRC. These were also reproduced in our study (Fig. 2C; Fig. S2). We noticed an obvious difference in study design. Almost all studies reported previously used the healthy volunteers as control, while we selected non-cancer patients as control. This may explain the poor reproducibility of what we identified compared with previous studies. The significantly changed metabolites included a lot of lipids in this study, which were rarely seen in previously studies. Interestingly, Janice Miller et al. also performed a study on patient-only cohort of upper GI cancer and identified a cluster of lysolipids which could discriminate the weight-loss patients (Miller et al., 2019b). Our cohort design is more in line with the real clinical scenario. For clinical diagnosis, the most challenging thing is discriminating the malignant and benign diseases. As reported by Feng Chen et al., the gut microbiome-associated metabolites in blood could classify the colorectal abnormal patients and healthy control but did not work for distinguishing patients with CRC and colorectal adenoma, which was because of the metabolic changes already happened at the adenoma stage (Chen et al., 2022). Another situation is that tumors usually occur in conjunction with other benign diseases, for example, lung cancer with comorbid pneumonia. These comorbidities are difficult to be accurately diagnosed clinically.

It is difficult to ensure the reproducibility of untargeted clinical metabolomics, as results can be easily influenced by individual and experimental factors, such as the cohort size, demographics, sample type and handling, analytical methods, and even data analysis, leading to a high level of inconsistency (Ren et al., 2021; Roth & Powers, 2022; Yu et al., 2020). These external factors can be addressed through consistent experimental design and normalized practices to achieve reliable and reproducible biomarker discovery. The inherent characteristics of a disease, however, enable us to decipher specific metabolic fingerprints, although reprogramed metabolism is a common cancer hallmark, such as in the Warburg-effect, which results in extremely low glucose and high lactate and glycolytic intermediate concentrations in tumor tissues (Hanahan & Weinberg, 2011; Hirayama et al., 2009). Indeed, the actual tumor microenvironments differ in organs or even in tissues, resulting in cellular heterogeneity at genetic and post-translation levels (Altschuler & Wu, 2010). In addition, even the gut microbiota composition is associated with the metabolic phenotype of GI cancer (Cani & Jordan, 2018; Lee et al., 2021; Ren et al., 2021). Consequently, cancer- and organ-specific metabolites can be considered potential biomarkers for the differential diagnosis of different types of cancer. In this study, although the metabolic patterns of GC and CC could not be adequately distinguished through classification (Fig. 2A), these two cancers lead to different degrees of influence on cellular metabolism (Fig. S1-2). This was also found by Atsuki Ikeda and co-workers. They compared the level of serum metabolites among three GI cancers (esophageal cancer, gastric cancer and colorectal cancer), and a total of 58 compounds were altered commonly in these three cancers when compared to healthy control but some of them exhibited cancer-specificity (Ikeda et al., 2012). For instance, lactic acid, glycolic acid, l-glutamic acid, and l-glutamine were significantly elevated in esophageal cancer and colorectal cancer; 3-hydroxypropionic acid and 3-hydroxyisobutyric acid were significantly increased in gastric and colorectal cancer; changes of malonic acid and l-serine were specific for esophageal cancer; 3-hydroxypropionic acid and pyruvic acid were contributed for separating gastric cancer; and l-alanine, glucuronoic lactone and l-glutamine characterized colorectal cancer. This was also deeply discussed in another review. Their hypothesis is that, on molecular level, genomic re-programming in each cancer type influences its metabolome (Ren et al., 2021). In other words, tumors arising in different tissue types may all have specific metabolite profiles to distinguish from one another. This difference may also be resulted from tumor microenvironment and microbiota. The well-reported bacterial metabolites (i.e., lactate, butyrate, acetaldehyde, and bile acids) play unignorable roles in different GI cancer (Bultman & Jobin, 2014; Nasr et al., 2020; Zeng et al., 2019; Zhao et al., 2020). There details indicated that metabolomics is useful for discovering the metabolic biomarkers of GI cancer, however, there is also full of challenges in distinguishing the GI cancer types. Recently, a metabolomics-based multicancer diagnosis study showed that the GC and CRC samples cannot be classified well on metabolic feature level (Zhang et al., 2022). This may also be due to their untargeted metabolomics with poor compound annotation. Based on the ROC analysis, the top five diagnostic metabolites of GC and CC were found to consist of three lipids and two other metabolites, but they were totally different in identify. In the comparison of GC and CC groups, there were also four lipids that contributed to classification, and the diagnostic results were satisfactory (Fig. 3). This suggests that metabolomics certainty has the potential to identify the type of GI cancer, or in other words, the organ of the primary tumor, which is not possible with genomic-based liquid biopsies (Cohen et al., 2018). Moreover, the more challenging thing is reproducing the cancer-specific biomarkers from previous studies with different platforms and self-developed compound libraries.

Tremendous efforts are being devoted to the discovery of diagnostic biomarkers for cancer, while prognostic studies are rarely conducted because of the limited samples and follow-up information (Wang et al., 2020). As metabolomics is a more direct reflection of an organism’s pathological and physiological states, it facilitates seeking the differential metabolites before and after surgery, which further assists us understand the surgical treatment mechanisms, promote the clinical management, and assess the outcome and risk of recurrence (Gao et al., 2023). The most representative study of metabolomics in clinical surgery is bariatric surgery, including Roux-en-Y gastric bypass (RYGB), sleeve gastrectomy (SG), etc. (Buchwald et al., 2004). For example, RYGB altered the metabolism of fatty acid, glucose, and amino acid, and SG caused the elevations of putrescine and several polyamines acetylated metabolites in serum (Lopes et al., 2015; Ocaña-Wilhelmi et al., 2020). Here a few studies that focused on the metabolic changes by surgery in gastric and colorectal cancer. The most influenced metabolic pathways were amino acid and lipid metabolism, and ascorbate and aldarate metabolism after surgical resection in GC and CRC patients (Jung et al., 2014; Lee et al., 2020; Vignoli et al., 2021; Zhuang et al., 2022). In this study, we detected the plasma metabolites after surgical treatment and performed a comparison with those before surgery from the identical subject. In the GC group, surgical resection dramatically altered the plasma metabolome, leading to a cluster of metabolites were significantly changed in the plasma (Fig. 5). Therein, (*S*)-(–)-cotinine and threonine reduced and l-lysyl-l-glutamine and threonic acid increased to normal level. In a urinary metabolomics study, alanine, arginine, and hypoxanthine were significantly changed after GC surgery and arginine returned to normal level after seven days (Jung et al., 2014). Although some longer intervals were observed in the CC group (i.e., 37, 40, and 42-day intervals), this did not contribute significantly to the differentiation of the metabolome before and after surgery. This may be a result of the metabolome being cancer-specific, the surgical operations or the patient-specific conditions. As Vignoli et al., stated, differentiated metabolites have statistically correlations with clinical variables (i.e., tumor size, tumor stage, tumor localization, and even patient gender) (Vignoli et al., 2021). There were also 10 metabolites that significantly changed by surgery and close to normal condition, namely, citric acid, beta-hydroxylauric acid, 3-hydroxytetradecanoic acid, l-histidyl-l-aspartic acid, 1,7-dimethyluric acid, (R)-2-aminobutanoic acid, l-aspartyl-l-valine, bavachinin, zinnimidine, and Cer d46:0 (Fig. 5B). The citric acid cycle plays a central role in the cellular energy metabolism and biosynthesis of macromolecules in mitochondrion. In tumor cells, the citric acid cycle switched the source input from glycolysis to additional energy sources (Pavlova & Thompson, 2016). Based on a population-level cohort study in UK, the genetic variants of the citric acid cycle was significantly associated with CRC, especially between the SUCLG2 gene rs35494829 and colon cancer (Cho et al., 2020). This indicates that the energy metabolism of tumor cells, especially the altered metabolic profile of the citric acid cycle processes the potential for colorectal cancer biomarker discovery (Cheng et al., 2012; Tan et al., 2013). As speculated, citric acid was significantly elevated in CRC samples (Amir Hashim et al., 2021; Tan et al., 2013; Uchiyama et al., 2017; Zamani et al., 2014). Besides, Buszewska-Forajta et al., found that citric acid was changed in prostate patient and have significant correlations with tumor progression (Buszewska-Forajta et al., 2022). In our study, the citric acid concentration was decreased after surgical resection, which is logical with the previous finding. There were two fatty acids with middle and long chains separately (beta-hydroxylauric acid, 3-hydroxytetradecanoic acid), which may indicate the fatty acid metabolic disorders in CRC patients (Brown et al., 2016; Chickos et al., 2002). In addition, we identified two exogenous metabolites, which may be originated from food, and several amino acids. Furthermore, the metabolites that were changed by surgery and still significantly different from NC controls (marker with * in Fig. 5) should be monitored for a longer period, as the postsurgical sampling time has a critical impact on metabolomics analysis. In conclusion, these significantly changed metabolites could be further validated for post-surgery monitoring.

The obvious limitations of this study are the small population of patients enrolled in each group, and the differences in the female percentage and smoking status between the groups, which might increase the bias of the findings. Therefore, the putative biomarkers for GC and CC detection and identification need to be reproduced in a larger cohort study on normalized baseline and further validated with independent cohorts. This study explored the possibility of metabolomics for GI cancer screening and tumor organ classification. Additional GI cancer types (i.e., belongs to GI tract and accessory organs of digestion, esophagus, stomach, small intestine, colon and rectum, liver, gallbladder, and pancreas) will be incorporated in the future to develop a complete diagnostic model for GI cancers with high sensitivity and specificity. In addition, the impact of chronic diseases and gut microbiota on metabolic perturbations and cancer-specific biomarker screening remains to be further elucidated. The metabolites that significantly changed because of surgical resection should also be validated in a study with a large sample size, and a longitudinal design is required with sufficient sampling time points and follow-up information to access the prognostic ability of the metabolite biomarkers.

## Conclusion

This case–control study was designed to explore the potential role of metabolomics for GI cancer detection and tissue-of-origin identification. Comparative metabolomics showed that the plasma metabolites differed between the cancer and non-cancer groups, which could strengthen the clinical diagnostic outcomes through complementation or even substitution. Blood-based untargeted metabolomics has the potential to be developed as a tool for the detection and localization of GI cancers and for post-surgical surveillance in an efficient and patient-friendly way.

## Supplementary Information

Below is the link to the electronic supplementary material.

Supplementary file 1 (DOCX 750 KB)

Supplementary file 2 (XLSX 89 KB)

Supplementary file 3 (XLSX 276 KB)

Supplementary file 4 (XLSX 32 KB)

Supplementary file 5 (XLSX 352 KB)

## Data Availability

The data underlying this article will be shared at reasonable request to the corresponding author.

## Abbreviations

GI: Gastrointestinal
GC: Gastric cancer
CC: Colon cancer
RC: Rectal cancer
CRC: Colorectal cancer
NC: Non-cancer
CT: Computerized tomography
MRI: Magnetic resonance imaging
TCA: The tricarboxylic acid
MTBE: Methyl-tert-butylether
MS: Mass spectrometry
GC–MS: gas chromatography-mass spectrometry
LC–MS: liquid chromatography-mass spectrometry
UPLC: Ultra-performance liquid chromatography
ROC: Receiver operating characteristic
AUC: Area under the ROC curve
TAG: Triglyceride
PE: Phosphatidylethanolamine
PC: Phosphatidylcholines
PG: Phosphatidylglycerol
SM: Sphingomyelin
Cer: Ceramides
