# Supplementary material for: Circulating metabolites as potential biomarkers for the early detection and prognosis surveillance of gastrointestinal cancers
Source: Metabolomics. 2023 Apr 4;19(4):36. doi: 10.1007/s11306-023-02002-0 (PMC10073066; doi:10.1007/s11306-023-02002-0)
Supplement: Supplementary file 1 — (DOCX 750 KB) [file 11306_2023_2002_MOESM1_ESM.docx]

**Circulating Metabolites as Potential Biomarkers for Early Detection and Prognosis surveillance in Gastric Cancer and Colon Cancer**

**Guodong Song^1*^, Li Wang^1*^, Junlong Tang^2^, Haohui Li^2^, Shuyu Pang^2^, Yan Li^2^, Li Liu^2#^, Junyuan Hu^2#^**

^1^ The Second Hospital of Tianjin Medical University, No 23. Pingjiang Road., Hexi District, 300211, Tianjin, China.

^2^ Metanotitia Inc., No 59. Gaoxin South 9th Road, Yuehai Street, Nanshan District, 518056, Shenzhen, Guangdong, China.

*Authors equally contributed to this study

# Corresponding author


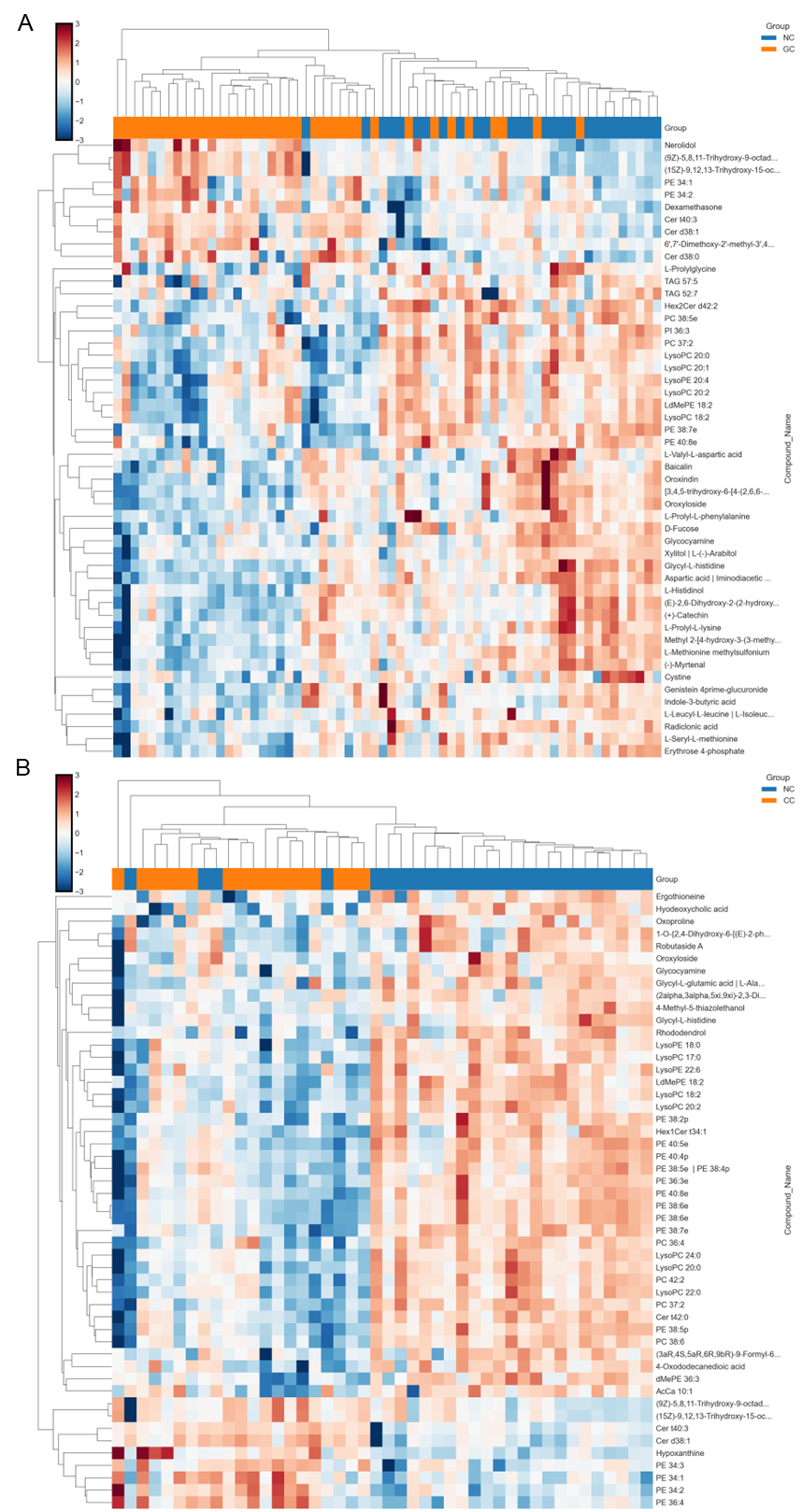


# Figure S1. Metabolomics differentiation of gastric and colon cancer compared with non-cancer samples.

Heatmap of top 50 metabolites altered between cancer and non-cancer groups. Cluster method: weighted. A. gastric cancer (GC) vs non-cancer (NC); B. colon cancer (CC) vs non-cancer (NC).


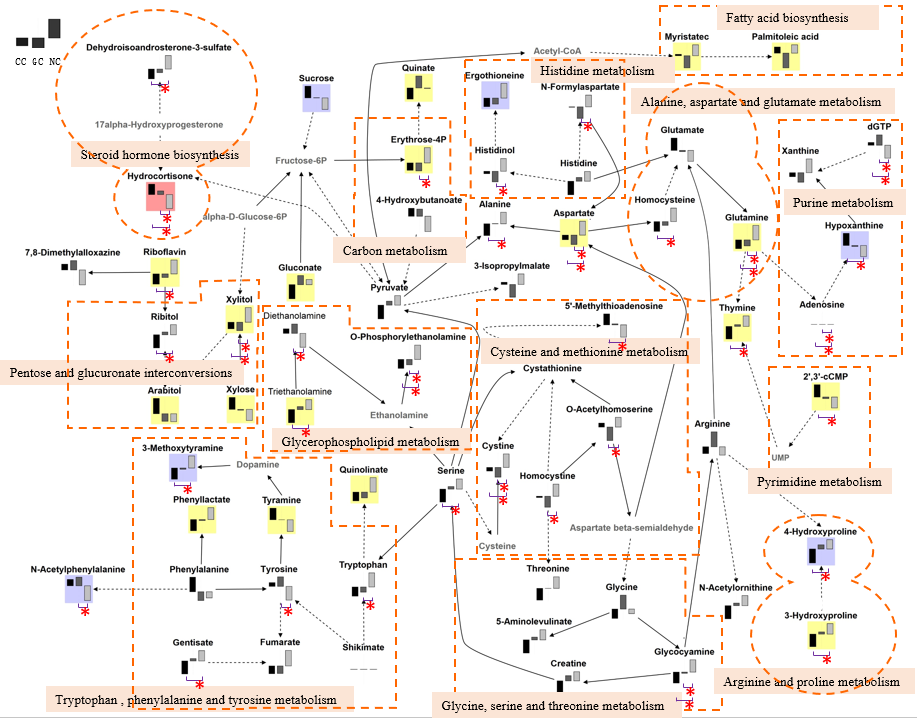


# Figure S2. Differential metabolic pathways of gastric and colon cancer compared with non-cancer samples.

The network was visualized by VANTED (<http://vanted.sourceforge.net/#ui-tabs-4>). Changed metabolites were screened by XGBoost (VIP>1). Undetected metabolites in this study were marked in grey. Yellow and blue fills indicate the metabolites screened from the model of GC/NC and CC/NC groups respectively; the red fill indicates metabolites screened from both two modes. * *p* < 0.05 (*t*-test). GC: gastric cancer; CC: colon cancer; NC: non-cancer.
